# Induction of hairy roots by various strains of *Agrobacterium rhizogenes* in different types of *Capsicum* species explants

**DOI:** 10.1186/1756-0500-7-414

**Published:** 2014-06-30

**Authors:** Nursuria Md Setamam, Norrizah Jaafar Sidik, Zainon Abdul Rahman, Che Radziah Che Mohd Zain

**Affiliations:** 1 Faculty of Applied Sciences, Universiti Teknologi MARA, 40450 Shah Alam, Selangor, Malaysia; 2 Faculty of Science and Technology, Academic Centre of Bioscience and Biotechnology, Universiti Kebangsaan Malaysia, 43600 Bangi, Selangor, Malaysia

**Keywords:** *Capsicum annuum*, *Capsicum frutescens*, *Agrobacterium rhizogenes*, Hairy root culture, Hairy root induction, Explant type

## Abstract

**Background:**

*Capsicum annuum* and *Capsicum frutescens*, also known as “chilies”, belong to the Solanaceae family and have tremendous beneficial properties. The application of hairy root culture may become an alternative method for future development of these species by adding value, such as by increasing secondary metabolites and improving genetic and biochemical stability compared with normal *Capsicum* plants. Therefore, in this research, different types of explants of both species were infected with various *Agrobacterium rhizogenes* strains to provide more information about the morphology and induction efficiency of hairy roots. After 2 weeks of *in vitro* seed germination, young seedling explants were cut into three segments; the cotyledon, hypocotyl, and radical. Then, the explants were co-cultured with four isolated *A. rhizogenes* strains in Murashige & Skoog culture media (MS) containing decreasing carbenicillin disodium concentrations for one month.

**Results:**

In this experiment, thick and short hairy roots were induced at all induction sites of *C. annuum* while thin, elongated hairy roots appeared mostly at wound sites of *C. frutescens.* Overall, the hairy root induction percentages of *C. frutescens* were higher compared with *C. annuum*. Hairy root initiation was observed earliest using radicles (1^st^ week), followed by cotyledons (2^nd^ week), and hypocotyls (3^rd^ week). Cotyledon explants of both species had the highest induction frequency with all strains compared with the other explants types. Strains ATCC 13333 and ATCC 15834 were the most favourable for *C. frutescens* while ATCC 43056 and ATCC 43057 were the most favourable for *C. annuum*. The interactions between the different explants and strains showed significant differences with *p*-values < 0.0001 in both *Capsicum* species.

**Conclusions:**

Both *Capsicum* species were amenable to *A. rhizogenes* infection and hairy root induction is recommended for use as an alternative explants in future plant-based studies.

## Background

*Capsicum annuum* and *Capsicum frutescens* also known as “chilies” belong to the Solanaceae family [1]. These species have tremendous economic value as vegetables crops and medicinal plants in numerous countries. *Capsicum* species have multiple usages as medicinal drugs for various diseases because of their analgesic, anti-inflammatory, antioxidant and anticancer properties [2,3]. These beneficial properties of *Capsicum* species usually come from their major secondary metabolites such as capsaicinoids, capsinoids, quercetin and luteolin [4-7]. Currently, one of the developing trends is hairy root culture techniques that enable high production of these secondary metabolites for extensive industrial applications [8-10].

In recent years, hairy root culture has been chosen as an alternative method for the development of crops and medicinal plants such as *Capsicum* species because of its many advantages [11,12]. Hairy root cultures have been proven to increase secondary metabolite levels in various plants including *Capsicum* species for industrial purposes [8,13,14]. Another advantage is the improvement of the plant system with better genetic and biochemical stability compared with normal plants [15-17].

One of the main factors that contribute to achieving hairy root induction is the type of explant used. Several studies on hairy roots in *Capsicum* species have used various explants such as hypocotyls [18], cotyledons [19], leaves [20] and mesophyll protoplasts [21]. In this research, different types of explants of both *C. annuum* and *C. frutescens* were infected with various *A. rhizogenes* strains. The objective of this research was to provide information on the establishment of hairy roots for both *C. annuum* and *C. frutescens* in term of morphology and induction efficiency.

## Results and discussion

### Effect on morphology of hairy root induction

In this experiment*,* young seedlings of *C. annuum* and *C. frutescens* germinated *in vitro* were used as study plants*.* Three types of explants—cotyledon, hypocotyl, and radical—from each *Capsicum* species were co-cultured with four isolated *A. rhizogenes* wild type strains (ATCC 15834, ATCC 43056, ATCC 13333, and ATCC 43057). After 1 month, hairy roots were induced by all *A. rhizogenes* strains for all explants types in both *Capsicum* species*.*

**Figure 1:**
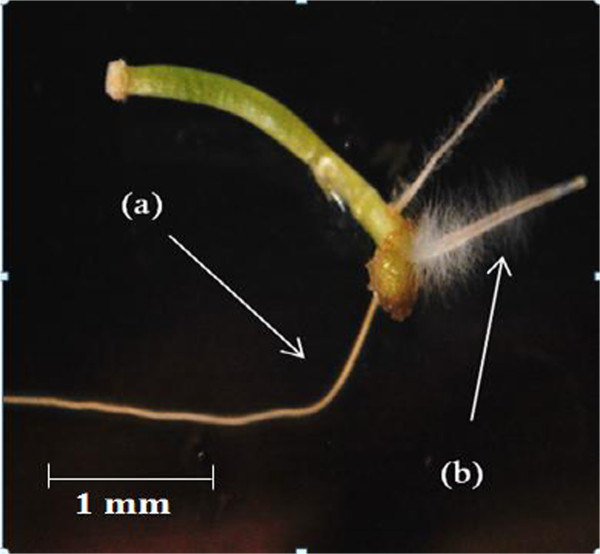
**Different root morphologies from explants of *Capsicum *** **spp. co-cultured with *A. rhzogenes*; (a) normal root, (b) hairy root.**

The successfully induced hairy roots of both *Capsicum* species appeared as whitish fungus needle-like structures (Figure 1b) compared with normal roots, which can be described as yellowish long rods with a smooth surface (Figure 1a). The normal roots of both *Capsicum* species appeared to follow geotropic patterns but the hairy roots showed a plagiotropic response. The plagiotropic growth of hairy roots in plants is possibly due to the lack of amyloplasts in the starch grains of hairy roots. Sensory balance receptors called statoliths lead to the diverse hairy root growth directions [22,23].

**Figure 2:**
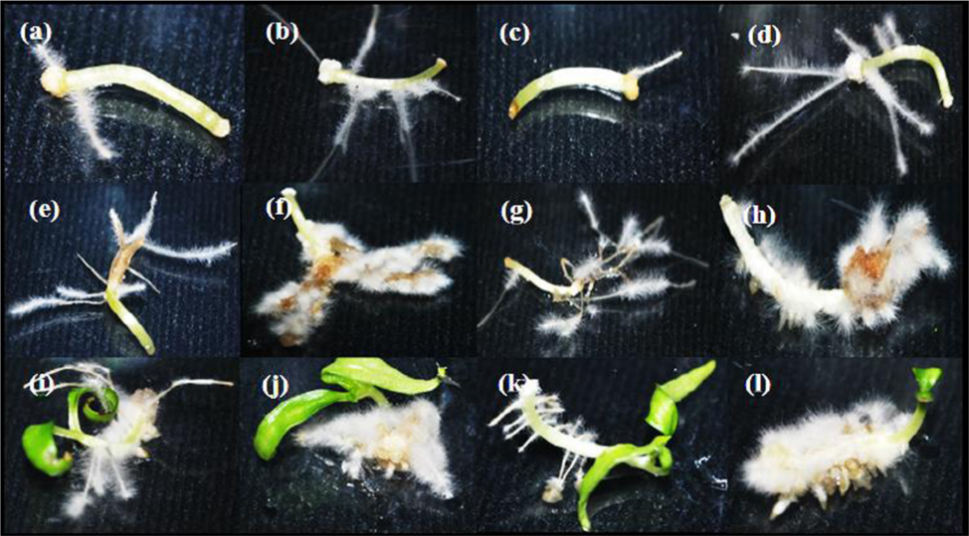
***Capsicum annuum *explants co-cultured with different *Agrobacterium rhizogenes *strains for one month. (a)** Hypocotyl with ATCC 15834, **(b)** hypocotyl with ATCC 43056, **(c)** hypocotyl with ATCC 13333, **(d)** hypocotyl with ATCC 43057, **(e)** radicle with ATCC 15834, **(f)** radicle with ATCC 43056, **(g)** radicle with ATCC13333, **(h)** radicle with ATCC 43057, **(i)** cotyledon with ATCC 15834, **(j)** cotyledon with ATCC 43056, **(k)** cotyledon with ATCC13333 and **(l)** cotyledon with ATCC 43057.

**Figure 3:**
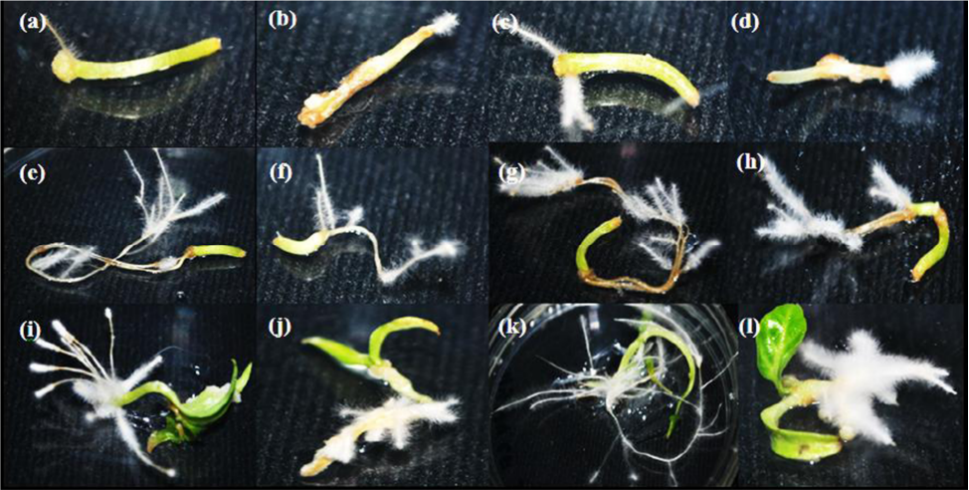
***Capsicum frutescens *explants co-cultured with different *A. rhizogenes *strains for 1 month. (a)** Hypocotyl with ATCC 15834, **(b)** hypocotyl with ATCC 43056, **(c)** hypocotyl with ATCC 13333, **(d)** hypocotyl with ATCC 43057, **(e)** radicle with ATCC 15834, **(f)** radicle with ATCC 43056, **(g)** radicle with ATCC13333, **(h)** radicle with ATCC 43057, **(i)** cotyledon with ATCC 15834, **(j)** cotyledon with ATCC 43056, **(k)** cotyledon with ATCC13333 and **(l)** cotyledon with ATCC 43057.

The induced hairy roots of both *Capsicum* species were similar in terms of structure, colouration, and growth pattern in each treatment. However, the length, thickness, and induction site varied depending on the type of strain and explant used. Thick and short hairy roots appeared on radicle and cotyledon explants treated with ATCC 43056 and ATCC 43057 in *C. annuum* (Figure 2), while all explants of *C. frutescens* showed thin, elongated hairy roots except in cotyledon explants treated with ATCC 43056 and 43057 (Figure 3).

Wound sites are a common location for hairy root induction since they serve as a genetic transfer point for *A. rhizogenes*[24,25]. In hypocotyl explants of both species, hairy roots were induced at the wound site except for hypocotyls of *C. annuum* with ATCC 43056 and ATCC 43057 (Figure 2). However, in radicle explants of both species, hairy roots were only induced far from the wound sites with the exception of *C. annum* with ATCC 13333 and ATCC 43057 (Figure 2). Additionally, hairy roots were induced at the wound sites of *C. frutescens* cotyledon explants by all strains (Figure 3).

The formation of callus in plant cultures is usually caused by additional plant hormones present in the medium. However, in hairy root cultures, the active expression of *rol* genes due to the presence *vir* genes on the *A. rhizogenes Ri*-plasmid may cause extreme synthesis of endogenous auxin and cytokinin in the host cells [26]. This may lead to the production of both hairy roots and callus simultaneously [27]. In our experiment, *C. annuum* was able to produce both callus and hairy roots at the same time, in contrast to *C. frutescens*. Both hypocotyl and radicle explants of *C. annuum* with strains ATCC 43056 and ATCC 43057 (Figure 2) were able to establish whitish, soft-textured callus at the wound sites. *C. annuum* treated with ATCC 15834 and ATCC 13333 (Figure 3) also produced callus at the wound sites of cotyledon explants.

Direct co-culturing with *A. rhizogenes* strains can result in bacteria residing in the plant cells [28,29]. However, active infection can cause cells to rupture and cell death known as necrosis. In *in vitro* cultures, the remaining necrotic cells may secrete phenolic compounds and fungal avirulence proteins that are highly toxic [30]. The presence of necrosis and antibiotics also may affect hairy root induction and contamination in samples [31,32].

### Effect on hairy root induction

In this experiment, three parameters were used to estimate the hairy root induction efficiencies of both *C. annuum* and *C. frutescens.* These parameters were hairy root induction percentage per total explants, hairy root initiation days per total explants and hairy root induction frequency per single explant.

Overall, the results showed that the hairy root induction percentages of *C. frutescens* were higher compared with *C. annuum*. Hypocotyl explants for both *Capsicum* species showed induction percentages of 55–75% with all strains. The mean percentages for *C. frutescens* radicle and cotyledon explants with all strain types were the highest at 100% hairy root induction.

**Figure 4:**
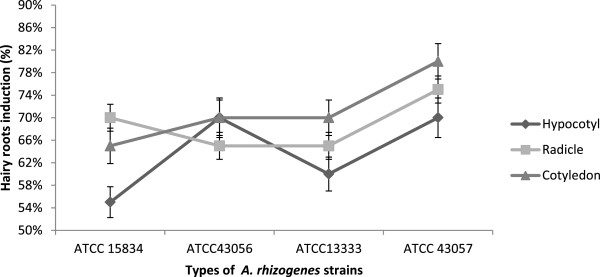
**Percentages of hairy root induction per total explants *(n =*** **20*) *of *Capsicum annuum *after 1 month.**

**Figure 5:**
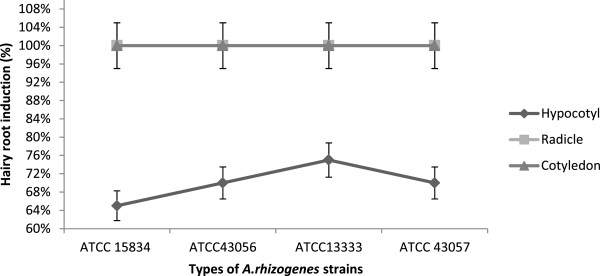
**Percentages of hairy root induction per total explants (*n*** **= 20) of *Capsicum frutescens *after 1 month.**

The radicles and cotyledons of *C. annuum* showed less hairy root induction with percentages between 65% and 80% for all strains. Cotyledon explants of *C. annuum* with ATCC 43057 had the highest induction rate at 80% (Figures 4 and 5). These results demonstrate that both explant type and species play a role in hairy root induction. This may be because each species expresses *rol* genes differently [25,33].

**Figure 6:**
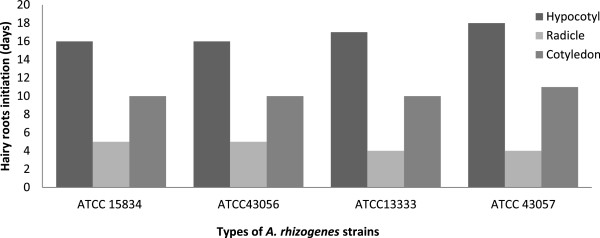
**Hairy root initiation (days) per total explants (*n*** **= 20) of *Capsicum annum *after 1 month.**

**Figure 7:**
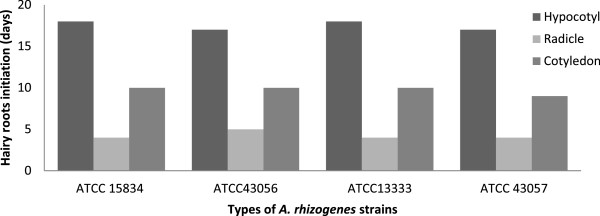
**Hairy root initiation (days) per total explants (*n*** **= 20) of *Capsicum frutescens *after 1 month.**

The earliest hairy root induction occurred within a week from a radicle of *C. frutescens* treated with ATCC 43057, while the longest period of time for hairy root induction was 18 days from hypocotyl explants with all strain treatments for both *Capsicum* species (Figures 6 and 7). The earliest hairy root initiation was observed in ascending order in radicles (1^st^ week), cotyledons (2^nd^ week), and hypocotyls (3^rd^ week). This was because of the properties of both radical and cotyledon explants, which consist of meristematic cells that are highly active in cell division [34,35].

Hairy roots were induced faster from radicle explants than cotyledon explants in both species*.* This is because of the ability of the radicle to initiate pericycle cells around the root for greater hairy root establishment. The cotyledon explants in both species had slower initiation times because the meristematic cells specifically accentuate the formation of new leaves and axillary buds [35].

The hypocotyl explants of *C. annuum* and *C. frutescens* in all strain treatments had the highest hairy roots initiation times (Figures 6 and 7), which may have been due to lower cell division activities. These inactive cells may require more time to regulate the cell toward decisive factors such plant hormones, turgor pressures and cyclic-dependent kinases (CDKs) for cell expansion and differentiation [36,37]. Therefore, hairy root initiation was deferred up to the third week.

In this study, the number of hairy roots present in a single explant was expressed as the frequency of hairy root induction. Cotyledon explants of both species had the highest frequency with all strains compared with other explant types. The highest frequency recorded was for cotyledon explants of *C. frutescens* with ATCC 15834 at 15.75 ± 0.67. The ATCC 15834 strain also showed the highest frequency in both hypocotyl and radicle explants of *C. frutescens* with1.70 ± 0.53 and 7.75 ± 0.85, respectively (Table 1). However, ATCC 15834 showed the lowest induction frequency in hypocotyl, radicle, and cotyledon explants of *C. annuum* with 1.40 ± 0.47, 12.05 ± 0.61 and 12.80 ± 0.79, respectively. ATCC 1333 showed the second lowest values after ATCC 15834 compared with the other strains (ATCC 43056 and ATCC 43057) with 2.15 ± 0.90, 10.32 ± 0.96 and 13.50 ± 0.49 (Table 1). These results showed that the induction frequencies of *C. annuum* were lower with ATCC 13333 and ATCC 15834 for all explant types while *C. frutescens* had relatively higher frequencies.

**Table 1:** **Hairy root induction frequency per single explant (mean ± standard deviation) in both *Capsicum *** **spp. after 1 month**

**List of species**	**Explant type**	**Strain**			
		**ATCC 13333**	**ATCC43056**	**ATCC15834**	**ATCC 43057**
*Capsicum annuum*	Hypocotyl	2.15 ± 0.90	2.45 ± 0.63	1.40 ± 0.47	2.75 ± 0.61
	Radicle	10.32 ± 0.96	13.20 ± 0.78	12.05 ± 0.61	13.35 ± 0.58
	Cotyledon	13.50 ± 0.49	14.05 ± 0.46	12.80 ± 0.79	13.8 ± 0.96
*Capsicum frutescens*	Hypocotyl	1.55 ± 0.68	1.15 ± 0.96	1.70 ± 0.53	1.45 ± 0.76
	Radicle	7.50 ± 0.69	6.75 ± 0.47	7.75 ± 0.85	7.00 ± 0.50
	Cotyledon	10.30 ± 0.82	7.70 ± 0.93	15.75 ± 0.67	9.45 ± 0.51

Thus, we concluded that ATCC 13333 and ATCC 15834 were the most favourable for *C. frutescens*, while ATCC 43056 and ATCC 43057 were the most favourable for *C. annuum*. The most suitable explants for a high frequency of hairy root induction in *C. annuum* and *C. frutescens* were the cotyledon and radicle. The explant types in *C. annuum* and *C. frutescens* showed interactions affecting the frequency of hairy roots per single explant with highly significant differences, with *p* < 0.0001 < < 0.05 (Tables 2 and 3). The different strains showed no significant differences in *C. annuum* (*p*-value = 0.8495 < 0.05) (Table 2) but had significant differences in *C. frutescens*, with *p* < 0.0001 (Table 3). The interactions between the different explant types and the different strains showed significant differences with *p*-values < 0.0001 in both *Capsicum* species (Tables 2 and 3). Therefore, the interactions between both explant type and bacterial strain can affect the frequency of hairy roots per single explant in both *Capsicum* species.

**Table 2:** **Two-way ANOVA (α = 0.05) for hairy root induction frequency per single explant of *Capsicum annum *** **after 1 month**

**Source of variation**	**Sum of squares (SS)**	**Degree of freedom (DF)**	**Mean square (MS)**	**F-statistic**	**Significant level (p-value)**
A: Types of explants	5944.633333	2	2972.316667	27.47	0.0001
B: Types of strains	86.512500	3	28.837500	0.27	0.8495
A × B	6031.14583	5	1206.22917	11.15	0.0001
Error	25318.65000	234	108.19936		
Corrected total	31349.79583	239			

**Table 3:** **Two-way ANOVA (α = 0.05) for hairy root induction frequency per single explant of *Capsicum frutescens *** **after 1 month**

**Source of variation**	**Sum of squares (SS)**	**Degree of freedom (DF)**	**Mean square (MS)**	**F-statistic**	**Significant level (p-value)**
A: Types of explants	3351.308333	2	1675.654167	80.37	0.0001
B: Types of strains	356.016667	3	118.672222	5.69	0.0009
A × B	3707.325000	5	741.465000	35.56	0.0001
Error	4878.658333	234			
Corrected total	8585.983333	239			

## Conclusions

In this study, both *C. annuum* and *C. frutescens* were amenable to *A. rhizogenes* infection. Both species showed positive responses, yielding hairy roots without the presence of exogenous plant hormones. These hairy roots exhibited whitish, fungus needle-like structures and plagiotropic growth similar to those in other plant studies. However, morphological variations were still seen between the two species in terms of length, thickness and the site of hairy root induction. Variations in species, strain and explant type led to different hairy root induction efficiencies. *C. annuum* was more amenable to ATCC 43056 and ATCC 43057 compared with ATCC 15834 and ATCC 13333, while *C. frutescens* was more amenable to ATCC 15834 and ATCC 13333 compared with ATCC 43056 and ATCC 43057. Despite the antagonistic response demonstrated by these two species, the various explant types showed similar results. In ascending order, the most suitable explant types for maximum hairy root induction were cotyledons, radicles and hypocotyls. Overall, our results suggest that hairy root induction could be used for alternative explants in future plant-based studies such as plant regeneration, somatic embryogenesis, molecular analysis or phytochemical studies.

## Method

### Seed sterilization and *in vitro* seed germination

Seeds of *C. annuum* var. *cayenne pepper* and *C. frutescens* var. *bird’s eye chili* were obtained from a local supplier. The seed edges were cut approximately 1 mm before soaking them in sterilised water for a day. Then, the seeds were washed under tap water for 5 minutes before the seed surfaces were sterilised in 15% sodium hypochlorite (NaClO) with two drops of Tween 80 for 10 minutes. The seeds were then rinsed twice with sterile water before being sterilised with 70% ethanol for 3 minutes. Finally, the seeds were rinsed again several times with sterile water. Ten sterilised seeds were cultured in individual Petri dishes that contained half-strength MS solid media. The seeds were left to germinate under a 16/8 h (light/dark) photoperiod for two weeks.

### Culture medium preparation

Full-strength MS medium was prepared by adding 4.4 g of MS powder, 1 g myo-inositol, and 30 g sucrose to 1 L of sterile distilled water. The solution pH was adjusted to approximately 5.7 to 6.0 using hydrochloric acid (HCl) or sodium hydroxide (NaOH). About 4 g gelrite was added to the medium before autoclaving at 121°C and 15 psi for 20 minutes. Half-strength MS solid medium was prepared similarly but the MS powder, myo-inositol and sucrose amounts were reduced by half.

### Preparation of sterile explants and pre-culturing

After 2 weeks of *in vitro* seed germination, 20 young seedling explants were cut into three segments (cotyledon, hypocotyl, and radicle) about 7–10 mm long. The cut segments were pre-cultured in Petri dishes containing half-strength MS solid medium for 1 day.

### Bacterial strains and cultures

Four wild *A. rhizogenes* strains (ATCC 15834, ATCC 43056, ATCC 13333 and ATCC 43057) were used for hairy root induction. The isolated strains were cultured in 0.01 L nutrient broth (NB) medium. The NB culture medium was prepared by weighing out 8 g/L NB powder and transferring it into 1 L of sterile water. Then, the solution was autoclaved and 0.01Lwas transferred into individual universal bottles. After three days, 10 μL of each isolated strain was inoculated into the NB cultures for three more days at a temperature of 26°C. The bacterial cultures with an optical density (OD) of 500–600 nm were used for co-culture after being shaken at 300 rpm for an hour. For culture preservation, 1mLeach of the main cultures were kept in 0.009 L nutrient broth at 10^−1^ dilution at a temperature of 26°C for not more than 1 week. The unused working cultures were stored in 20% glycerol and kept frozen in −20°C freezer.

### Co-culture and hairy root culture establishment

Each explant was immersed in 10 μL of the isolated strains in Petri dishes containing half-strength MS solid medium for a day. Then, the co-cultured explants were decontaminated using washing medium containing MS liquid medium with carbenicillin disodium (1 g/L) for 2 hours. The co-cultured explants were dried with sterile filter paper to remove excess bacteria before culture on full-strength MS solid medium containing 0.5 g/L carbenicillin disodium. The explants were sub-cultured each week on MS solid medium containing decreasing carbenicillin disodium concentrations (0.2, 0.1, 0.05 mg/L) for 1 month. Explants without *A. rhizogenes* strain treatment were used as controls. The experiments were performed with 20 replications per each treatment (*n* = 20).

### Statistical analysis

The data were collected after one month and analysed using a two-way analysis of variance (ANOVA) with SAS 9.0 and standard deviation mean values expressed as mean ± SD.

## Competing interests

The authors have no competing interests to declare.

## Authors’ contributions

NMS carried out the overall research experiment, was involved in design of the study, performed statistical analysis, acquired and interpreted data, and prepared manuscript. NJS contributed to the overall design of the research study, coordinated the research experiment, and was involved in drafting the manuscript and revising it critically for important intellectual content. ZAR participated in design of the study involving microbiology and revising the manuscript critically for intellectual content. CRCMZ performed bacterial strain culture and was involved in maintaining bacterial culture stocks in the early stages of the research experiment. All authors have read and approved the final manuscript for submission.
